# Duration of immobilisation after Achilles tendon rupture repair by open surgery: a retrospective cohort study

**DOI:** 10.1186/s13018-021-02342-4

**Published:** 2021-03-17

**Authors:** Xiang Yu Xu, Shan Gao, Yang Lv, Fang Zhou, Chen Jiao, Ji Xing Fan, Teng Jiao Zhu

**Affiliations:** 1 Orthopaedic Department, Peking University Third Hospital, Haidian District, Beijing, 100191 China; 2 Department of Sports Medicine, Peking University Third Hospital, Haidian District, Beijing, 100191 China

**Keywords:** Achilles tendon rupture, Open surgery, Early rehabilitation, Immobilisation duration

## Abstract

**Background:**

The best treatment for acute Achilles tendon ruptures remains controversial. No cohort studies have compared different immobilisation durations after open surgery. This retrospective cohort study aimed to determine the optimal duration of immobilisation after this surgery.

**Methods:**

A total of 266 patients with acute Achilles tendon rupture were divided into 4 groups (A, B, C, and D) according to immobilisation duration of 0, 2, 4, and 6 weeks, respectively. All patients underwent the same suture technique with a similar rehabilitation protocol and were examined clinically at 2, 4, 6, 8, 10, 12, 14, 16, 24, and 48 weeks, with a final follow-up at a mean of 22.3 months postoperatively. The primary outcome was the time of return to light sports activity (LSA). Secondary outcomes included range of motion (ROM) and single-legged heel rise height (SHRH). Data on operation time, complications, visual analogue pain scale (VAS), American Orthopaedic Foot and Ankle Society (AOFAS) hindfoot score, and Achilles tendon Total Rupture score (ATRS) were also collected. Demographic baseline data were analysed using one-way analysis of variance; outcome parameters were analysed using Kruskal-Wallis *H* test, and complications were analysed using Fisher’s exact test. Statistical significance was considered at *P* ≤ 0.05.

**Results:**

VAS scores decreased significantly, reaching 0 in all groups after 12 weeks. The AOFAS and ATRS scores were significantly different between the groups from weeks 2 to 12 (*P<*0.001) and weeks 2 to 16 (*P<*0.001), respectively. All the mean scores showed better results in group B than in the other groups. In terms of recovery time of ROM, SHRH, and LSA, groups A and B were significantly faster than groups C and D (*P<*0.001). There were 13 (13/266, 4.9%) complications: 5 superficial infections, 3 deep venous thrombosis, and 5 trauma-related re-ruptures. On the last follow-up, all complications had recovered. There were no significant differences in complications between the groups.

**Conclusions:**

Immobilisation for 2 weeks after this open surgery is the best choice for early rehabilitation and weight-bearing while minimising pain and other complications.

## Background

Achilles tendon rupture (ATR) is a common injury with an increasing incidence. It most frequently occurs in 30–50-year-old men who periodically participate in recreational sports [1, 2] such as badminton (42%), volleyball (18%), soccer (10%), tennis (8%), or indoor hockey (6%) [3]. It occurs predominately in men, with a reported male-to-female ratio ranging from 2:1 to 12:1 [4, 5]. The best treatment for acute ATR remains controversial [6]. Although many researchers support nonoperative treatment [7, 8], surgical treatment may be advantageous, especially for athletes, for young people, and for those with chronic rupture [9].

Different suture techniques in open surgery have been reported [10–12]. However, there is no single uniformly accepted method for Achilles reattachment. Early functional mobilisation and weight-bearing exercises are increasingly being used to promote tendon healing [13–15]. Kangas et al. [16] have shown that patients who undergo early restricted functional treatment for 6 weeks postoperatively demonstrate improved isokinetic calf muscle strength. Mandelbaum et al. [17] showed that full weight-bearing is allowed in 2 to 3 weeks after strengthening of the suturing.

Many researchers have reported that conservative treatment can cause complications such as increased pain, re-rupture, calf muscle weakness, and delayed return to work [18–20]. To repair the Achilles tendon surgically, various methods, from open repair to limited open repair or percutaneous repair, have been reported [21, 22]. With standard methods of surgical treatment, patients frequently undergo limited weight-bearing, which often includes immobilisation using a brace for at least 6 weeks [23]. Early full weight-bearing is thought to increase the risk of re-rupture; however, early functional rehabilitation and weight-bearing results in the best functional recovery and shortens the rehabilitation time [13, 24].

Consequently, we consider that strengthening the surgical suturing and starting weight-bearing exercises as soon as possible after the operation may improve the prognosis for ATR. Therefore, it is important to safely shorten the immobilisation duration when starting early functional rehabilitation such that patients can go back to work or normal life as soon as possible. The purpose of this cohort study was to determine the optimal duration of immobilisation after open surgery to repair an ATR.

## Methods

### Study design

This retrospective cohort study aimed to determine the optimal duration of immobilisation after open surgery to repair an ATR. This study compared the treatment outcomes of different immobilisation times by dividing patients into 4 groups—A, B, C, and D—based on the amount of immobilisation time (0, 2, 4, and 6 weeks, respectively) that included a similar rehabilitation protocol.

A total of 315 patients who underwent ATR surgery at our university hospital between June 2015 and June 2019 were included in the study. Of these, 49 patients were lost to follow-up because they could not be contacted or did not complete the rehabilitation protocol. A total of 266 patients (84.4%) were included in the final analysis.

The inclusion criteria were patients aged 18 to 60 years with an acute closed single-legged complete ATR who underwent open surgery with our suture technique. We excluded patients with prior ATR and those without rehabilitation or follow-up outcomes. Other situations that affected patients’ lower limb functions or tendon healing (e.g. autoimmune disease, diabetes mellitus, systemic corticosteroid treatment) were also excluded.

### Operative technique

**Fig. 1:**
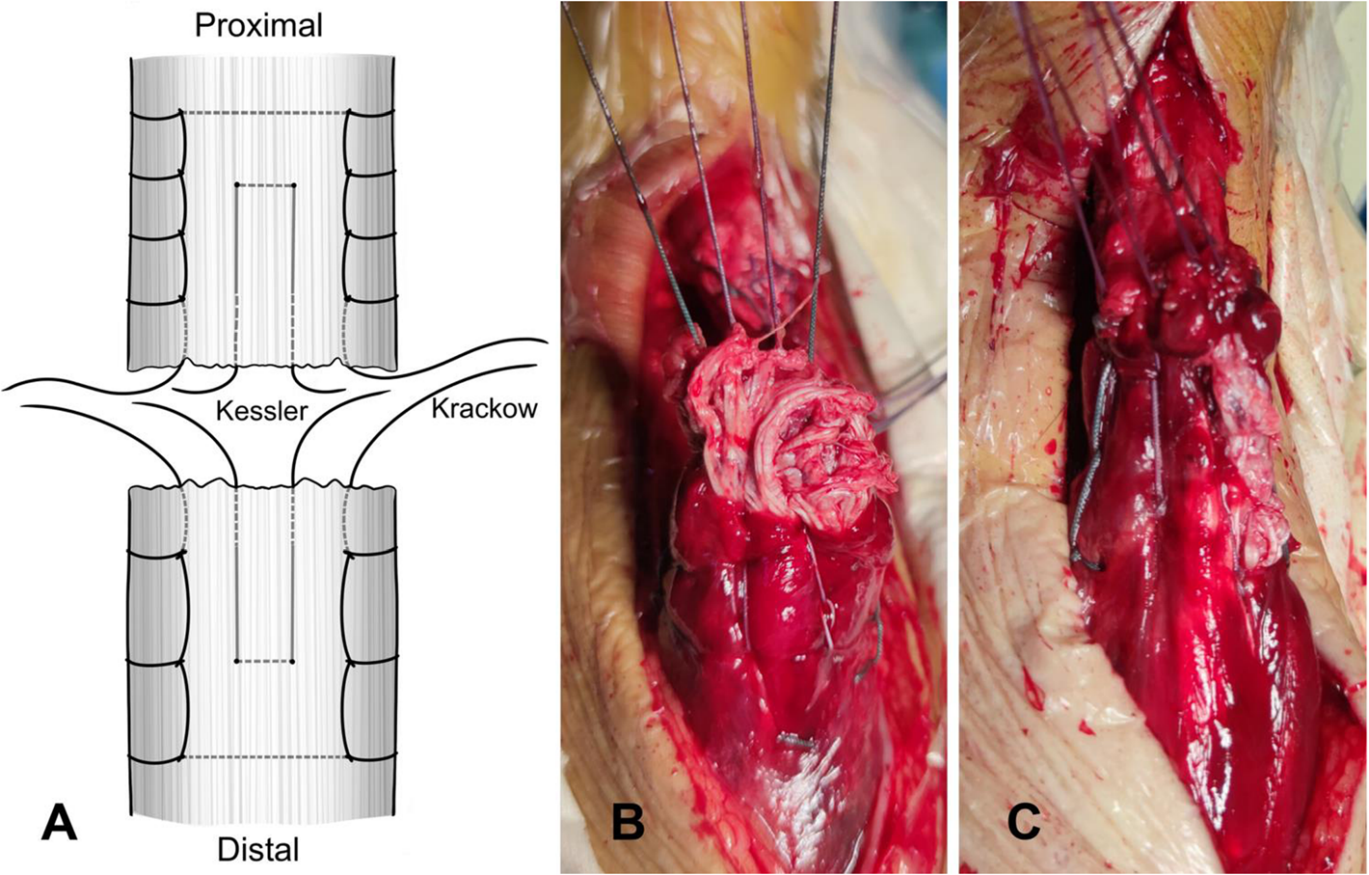
**a** Diagram of our suture technique. **b** Krackow locking loop technique combined with the modified Kessler technique. **c** Four figure-eight sutures for the dorsal to reinforce the broken ends

All patients included in the study underwent the same operative technique performed by the same surgeon. Patients were operated under spinal anaesthesia in a prone position using a tourniquet. A longitudinal posteromedial incision was made over the rupture site, and the paratenon was divided to identify the rupture. With the ankle placed in a neutral position, the tendon was repaired by the Krackow locking loop technique with 2 W4843 (ETHIBOND of Johnson & Johnson, USA) nonabsorbable sutures, and the modified Kessler suture technique with 2 REF223114 (ORTHOCORD of Depuy Synthes, USA) nonabsorbable sutures. The sutures were carefully placed away from the rupture site and sutured in the healthy tendon to enhance the stability of the repair (Fig. 1). Then, 6 to 8 (4 for the dorsal, 2–4 for the ventral) figure-eight sutures were made with 2-0-gauge absorbable sutures to reinforce the broken ends. The Thompson sign was positive after the suturing. After testing the tension and strength of the ankle, the fascia was then carefully resutured, and the skin was closed with a skin stapler. After binding the wound, a below-knee brace was applied with the ankle in a neutral position in all patients except for those in group A.

### Postoperative protocol

All patients were divided into 4 groups according to the duration of immobilisation. Group A included 42 patients (15.8%) with no immobilisation after the surgery. Group B included 89 patients (33.5%) with 2 weeks of immobilisation, group C included 83 patients (31.2%) with 4 weeks of immobilisation, and group D included 52 patients (19.5%) with 6 weeks of immobilisation postoperatively. Patients from group D were operated on earlier based on our prior protocol; we gradually shortened the immobilisation period afterwards so that group A was the last to be included in the study.

Patients in all groups were instructed to perform postoperative exercises on their own after the immobilisation brace was removed, following a suggested rehabilitation protocol (Table 1). Crutches were suggested for all the patients to help them do partial weight-bearing standing and early walking. Heel wedge was not used for all the patients after the brace removal.

**Table 1:** Rehabilitation schedule

	Group A (no IM)	Group B (2-week IM)	Group C (4-week IM)	Group D (6-week IM)
Ankle mobilisation	All the time	>2 weeks	>4 weeks	>6 weeks
Standing up for 1 h per day	0–2 weeks	2–4 weeks	4–6 weeks	6–8 weeks
Standing up for 2 h per day	2–4 weeks	4–6 weeks	6–8 weeks	8–10 weeks
Deep squat	2–4 weeks	4–6 weeks	6–8 weeks	8–10 weeks
Double-legged heel raises	4–6 weeks	6–8 weeks	8–10 weeks	10–12 weeks
Walking<1000 steps, no climbing stairs	4–6 weeks	6–8 weeks	8–10 weeks	10–12 weeks
Single-legged heel raises	6–8 weeks	8–10 weeks	10–12 weeks	12–14 weeks
Walking<3000 steps, no climbing stairs	6–8 weeks	8–10 weeks	10–12 weeks	12–14 weeks
Jogging	+2 weeks	+2 weeks	+2 weeks	+2 weeks
Vigorous activity recovery training	+4 weeks	+4 weeks	+4 weeks	+4 weeks

*IM* immobilisation

They did not receive any professional physical therapy throughout their rehabilitation. At 0–2 weeks after the brace was removed, patients were instructed to perform moderate plantar flexion and dorsiflexion of the ankle, with or without manual help. They were also instructed to stand up (partial weight-bearing) for 1 h every day. At 2–4 weeks after the brace was removed, the standing up time was increased to 2 h per day, and the ankle exercise remained the same. Patients were instructed to attempt deep squat exercises at 2–4 weeks. At 4–6 weeks after brace removal, patients were instructed to perform double-legged heel raises and to walk less than 1000 steps without climbing stairs. At 6–8 weeks after brace removal, patients were instructed to perform single-legged heel raises and to walk less than 3000 steps without climbing stairs. When the patients were able to successfully perform single-legged heel raises, they were instructed to perform jogging 2 weeks later. Four weeks after successful performance of jogging, patients were allowed to perform vigorous activity recovery training.

### Follow-up

The patients were examined clinically at 2, 4, 6, 8, 10, 12, 14, 16, 24, and 48 weeks, with a final follow-up at a mean of 22.3 (range, 12–43) months. The primary outcome was the time of return to light sports activity (LSA). LSA included rapid walking and jogging. Secondary outcomes included the range of motion (ROM) recovery time (dorsiflexion and plantarflexion) and the single-legged heel rise height (SHRH) recovery time. The operation time and complications, such as re-rupture, superficial infection, and deep venous thrombosis (DVT), were recorded. For an assessment of the subjective pain scale and functional status, the visual analogue scale (VAS) pain score, American Orthopaedic Foot and Ankle Society (AOFAS) hindfoot score, and the Achilles tendon Total Rupture Score (ATRS) were collected [25, 26]. For the VAS scores, we asked all the patients to grade their pain score according to the resting state, to suppress the interference of different rehabilitation exercise schedules for the 4 groups. The VAS scale is a 10-cm line with statements on the left (no pain) and on the right (worst possible pain). The patients mark their current pain level on the line. The AOFAS score ranges from 0 to 100, with a healthy hindfoot receiving 100 points. The ATRS is a patient-reported evaluation scale that measures outcomes related to symptoms and physical activity after treatment for total ATR. The ATRS includes 10 items; each item has a score ranging between 0 and 10 on a Likert scale, with 100 indicating no major limitations. Dorsiflexion and plantarflexion were measured with a handheld goniometer. The recovery time was recorded when the ROM was similar to that of the uninjured side. The heel rise height was measured as the distance from the ground to the heel when the patient lifted the heel while keeping the knee straight. The recovery time was noted when the SHRH was similar to that of the opposite leg.

### Statistical analysis

Statistical analyses were performed with the Statistical Package for the Social Sciences (SPSS) for Windows (v 25.0; IBM Corp). Data are reported as mean ± standard error of the mean unless otherwise noted. Demographic baseline data were analysed using one-way analysis of variance; outcome parameters were analysed using Kruskal-Wallis *H* test, and complications were analysed using Fisher’s exact test. Statistical significance was considered at *P* ≤ 0.05.

The study was approved by the regional Ethics Committee (IRB00006761-M2020252), and the need for informed consent was waived with the agreement of the Ethics Committee.

## Results

The series consisted of 262 males and 4 females (mean age, 35 years; range, 24–55 years). The majority (97%) sustained their rupture during sports-related activities, including basketball (116 patients, 43.6%), badminton (98 patients, 36.8%), soccer (26 patients, 9.8%), running (10 patients, 3.8%), and long jump (8 patients, 3.0%). No significant differences were found between the groups with regard to sex, age, body mass index, distance from the rupture site to Achilles tendon insertion site, gap distance of the rupture site, and operation time (Table 2).

**Table 2:** Patient characteristics

Variable	Group A (*n*=42)	Group B (*n*=89)	Group C (*n*=83)	Group D (*n*=52)	Total	*P* value
Men/women	40/2	88/1	83/0	51/1	262/4	
Age	36.1 ± 5.9	34.7 ± 5.4	35.4 ± 5.8	34.9 ± 4.2	35.2 ±5 .4	0.503
BMI	23.6 ± 1.9	24.4 ± 1.6	23.9 ± 2.6	23.4 ± 2.4	24.3 ± 2.3	0.547
DSTI	5.2 ± 0.9	5.1 ± 0.8	5.2 ± 0.8	5.3 ± 0.5	5.2 ± 0.8	0.473
GD	2.2 ± 1.0	2.2 ± 1.0	2.1 ± 1.0	2.0 ± 1.0	2.1 ± 1.0	0.838
OT	31.7 ± 4.8	31.7 ± 4.7	31.9 ± 4.6	32.2 ± 5.0	31.9 ± 4.7	0.947

Values are expressed as mean ± standard error of the mean

*BMI*, body mass index; *DSTI*, the distance from rupture site to Achilles tendon insertion; *GD*, the gap distance of rupture site; *OT*, operation time

### Outcome scores

**Fig. 2:**
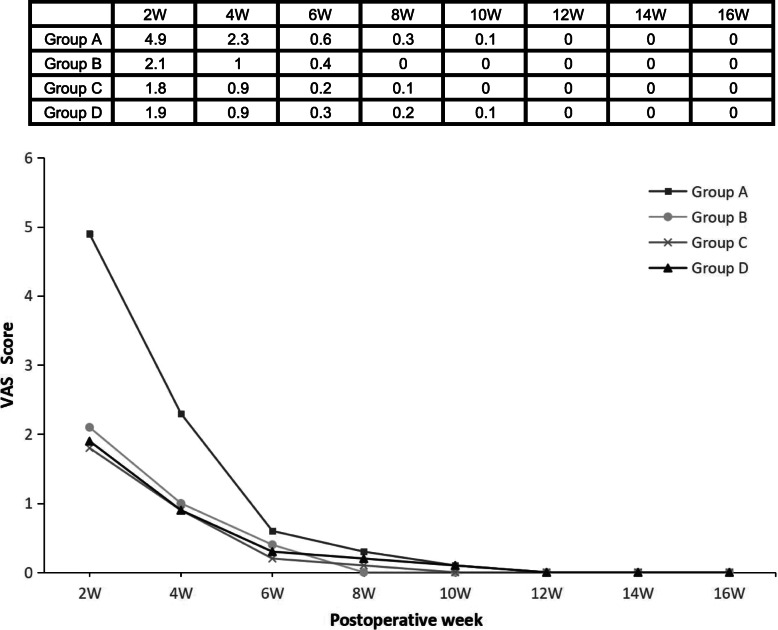
Mean visual analogue scale (VAS) pain score at each time point

From 2 to 10 weeks, the VAS scores decreased significantly with time, and after 12 weeks, the scores reached 0 in all groups. Significant differences were found among the 4 groups at 2 (*P<*0.001, group A showed a difference compared with the other groups), 4 (*P<*0.001, group A showed a difference compared with the other groups), 8 (*P =* 0.006, group B showed a difference compared with group D), and 10 weeks (*P<*0.001, group A showed differences from groups B and C) (Table 3, Fig. 2).

**Table 3:** Outcome scores and recovery time

	Group A (*n*=42)	Group B (*n*=89)	Group C (*n*=83)	Group D (*n*=52)	*P* value
VAS					
2 wk	4.9 ± 1.0	2.1 ± 1.0	1.8 ± 0.8	1.9 ± 0.6	<0.001
4 wk	2.3 ± 1.6	1.0 ± 1.1	0.9 ± 0.6	0.9 ± 0.6	<0.001
6 wk	0.6 ± 1.2	0.4 ± 0.9	0.2 ± 0.6	0.3 ± 0.7	0.431
8 wk	0.3 ± 0.8	0	0.1 ± 0.3	0.2 ± 0.5	0.006
10 wk	0.1 ± 0.3	0	0	0.1 ± 0.2	<0.001
AOFAS					
2 wk	50.6 ± 4.3	53.0 ± 4.7	56.1 ± 3.3	54.8 ± 1.6	<0.001
4 wk	69.4 ± 7.3	67.1 ± 7.8	61.9 ± 4.6	58.8 ± 5.6	<0.001
6 wk	88.2 ± 11.3	90.3 ± 10.8	82.8 ± 5.4	72.5 ± 5.6	<0.001
8 wk	90.6 ± 7.7	97.1 ± 6.1	90.1 ± 6.6	82.8 ± 5.2	<0.001
10 wk	95.0 ± 5.4	99.1 ± 2.0	97.0 ± 4.9	94.7 ± 6.1	<0.001
12 wk	97.6 ± 4.3	99.1 ± 2.0	98.5 ± 3.0	97.6 ± 4.3	<0.001
14 wk	98.4 ± 3.4	99.3 ± 1.7	99.6 ± 1.2	99.5 ± 2.1	0.060
16 wk	98.4 ± 3.4	99.7 ± 0.7	99.6 ± 1.2	99.8 ± 1.0	0.072
24 wk	99.0 ± 1.9	99.8 ± 0.5	99.7 ± 0.7	99.8 ± 0.6	0.072
48 wk	99.4 ± 1.2	99.8 ± 0.5	99.8 ± 0.7	99.9 ± 0.4	0.084
ATRS					
2 wk	9.9 ± 3.4	9.8 ± 1.9	8.0 ± 2.5	9.9 ± 2.0	<0.001
4 wk	32.5 ± 5.3	28.1 ± 6.9	16.6 ± 2.6	13.3 ± 3.0	<0.001
6 wk	50.0 ± 7.2	50.7 ± 7.4	40.9 ± 3.8	20.4 ± 5.8	<0.001
8 wk	67.8 ± 10.4	68.3 ± 6.0	57.4 ± 3.3	43.4 ± 4.8	<0.001
10 wk	74.0 ± 7.6	80.9 ± 5.7	78.2 ± 6.3	67.9 ± 4.7	<0.001
12 wk	82.0 ± 5.7	90.5 ± 5.3	86.8 ± 4.9	79.4 ± 4.3	<0.001
14 wk	90.5 ± 2.9	95.3 ± 3.0	91.4 ± 2.9	87.3 ± 3.8	<0.001
16 wk	96.4 ± 2.4	97.3 ± 1.3	95.0 ± 2.0	93.5 ± 2.3	<0.001
24 wk	97.1 ± 1.3	97.4 ± 1.0	96.9 ± 1.2	97.1 ± 1.3	0.082
48 wk	97.3 ± 1.0	97.4 ± 0.8	97.1 ± 1.0	97.4 ± 1.2	0.143
RT (weeks)					
ROM	6.6 ± 2.2	6.5 ± 1.7	8.6 ± 1.3	10.3 ± 1.1	<0.001
SHRH	12.6 ± 1.3	12.5 ± 1.9	14.2 ± 1.3	14.5 ± 1.5	<0.001
LSA	18.7 ± 2.0	18.3 ± 1.8	19.8 ± 1.4	20.4 ± 1.8	<0.001

Values are expressed as mean ± standard error of the mean

*VAS*, visual analogue scale; *AOFAS*, American Orthopaedic Foot and Ankle Society hindfoot score; *ATRS*, Achilles tendon Total Rupture Score; *RT*, recovery time; *ROM*, range of motion; *SHRH*, single-legged heel rise height; *LSA*, light sports activity

**Fig. 3:**
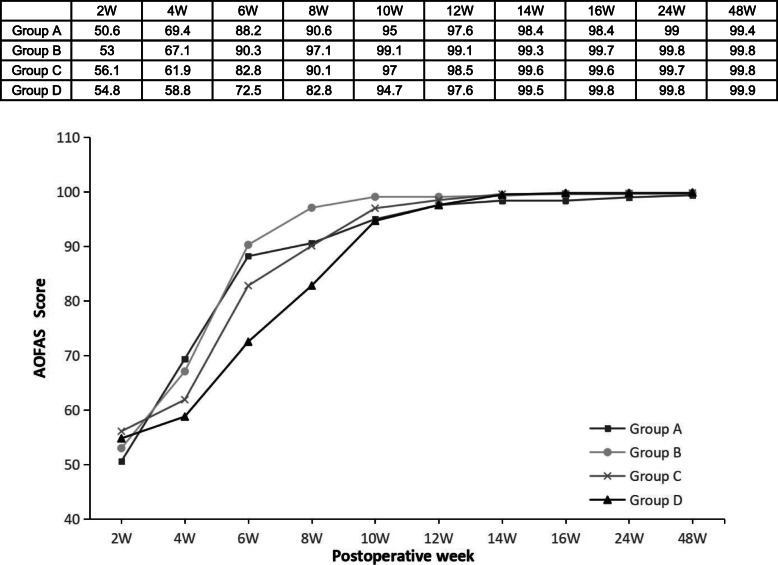
Mean American Orthopaedic Foot and Ankle Society (AOFAS) hindfoot score at each time point

The mean AOFAS scores in all groups increased with time. Regarding these scores, group B showed significant differences compared to the other groups: groups A (*P<*0.001) and C (*P=*0.013) at 2 weeks; groups C and D at 4 and 6 weeks (*P<*0.001); groups A, C, and D at 8 and 10 weeks (*P<*0.001); and group D at 12 weeks (*P<*0.001). There were significant differences among the 4 groups from weeks 2 to 12 (*P<*0.001, Table 3), and the mean scores of group B were higher than those of the other groups (Fig. 3).

**Fig. 4:**
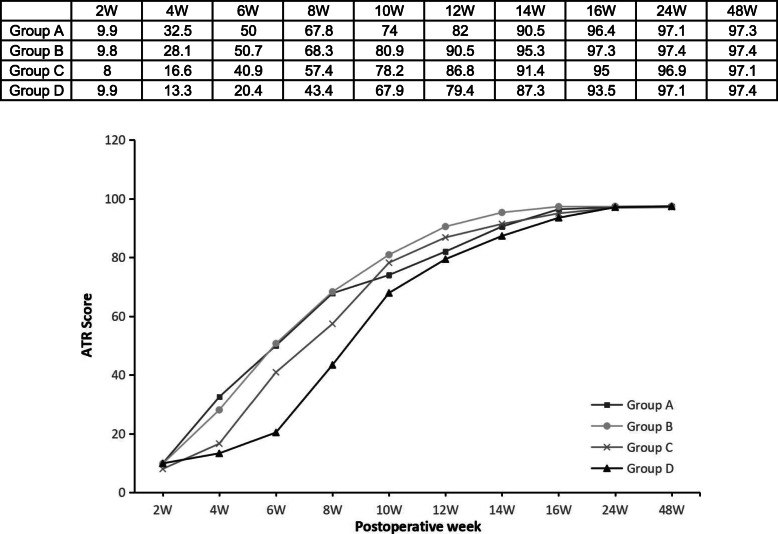
Mean Achilles tendon Total Rupture Score (ATRS) at each time point

The mean ATRS in all groups increased with time. Regarding these sores, group B showed significant differences compared to the other groups: group C (*P<*0.001) at 2 weeks; groups C and D at 4, 6, and 8 weeks (*P<*0.001); groups A (*P<*0.001), C (*P=*0.002), and D (*P<*0.001) at 10 weeks; groups A, C, and D at 12 and 14 weeks (*P<*0.001); and groups C and D at 16 weeks (*P<*0.001). Significant differences were found among the 4 groups from weeks 2 to 16 (*P<*0.001, Table 3), and the mean scores of group B were higher than those of the other groups (Fig. 4).

### Recovery time

The mean ROM recovery time was 6.6±2.2 weeks after the surgery in group A, 6.5±1.7 weeks in group B, 8.6±1.3 weeks in group C, and 10.3±1.1 weeks in group D. ROM recovery occurred significantly faster for groups A and B than for groups C and D (*P*<0.001, Table 3). The mean time to recover SHRH was 12.6±1.3, 12.5±1.9, 14.2±1.3, and 14.5±1.5 weeks in groups A to D, respectively. The SHRH for groups A and B was significantly different compared to that for groups C and D (*P*<0.001). The mean time to return to LSA was 18.7±2.0, 18.3±1.8, 19.8±1.4, and 20.4±1.8 weeks in groups A to D, respectively. Groups A and B also had significantly faster return to LSA than groups C and D (*P*<0.001).

### Complications

There were 13 (13/266, 4.9%) complications that occurred in this series, including 5 (5/42, 11.9%) in group A, 3 (3/89, 3.4%) in group B, 2 (2/83, 2.4%) in group C, and 3 (3/52, 5.8%) in group D. Two superficial infections occurred in group A, and one occurred in the other 3 groups (*P=*0.413). One DVT occurred in groups A, B, and D (*P=*0.451). There were 5 patients with re-rupture (2 in group A, one in the remaining groups) (*P=*0.413) that were diagnosed within 8 weeks after the operation. All 5 patients sustained the re-rupture during a fall or similar sudden trauma. At the last follow-up, all patients with complications had recovered without any problems. There were no significant differences in complications among the 4 groups (Table 4).

**Table 4:** Complications

Variable (%)	Group A (*n*=42)	Group B (*n*=89)	Group C (*n*=83)	Group D (*n*=52)	Total	*P* value
SF	2 (4.8%)	1 (1.1%)	1 (1.2%)	1 (1.9%)	5 (1.9%)	0.413
DVT	1 (2.4%)	1 (1.1%)	0	1 (1.9%)	3 (1.1%)	0.451
Re-rupture	2 (4.8%)	1 (1.1%)	1 (1.2%)	1 (1.9%)	5 (1.9%)	0.413
Total	5 (11.9%)	3 (3.4%)	2 (2.4%)	3 (5.8%)	13 (4.9%)	0.125

*SF*, superficial infection; *DVT*, deep venous thrombosis

## Discussion

In our study, most patients were recreational athletes, and 97% sustained their injury during sports-related activities. In decreasing order of frequency, they were injured during basketball (43.6%), badminton (36.8%), soccer (9.8%), running (3.8%), and long jump (3.0%); this is similar to the findings of other studies [3, 4, 27]. Notably, there were only 4 female patients in our study (male-female ratio was 65.5:1). This result could be attributed to the females’ preference for non-confrontational sports with a lower morbidity of the ATR. Further, female patients may be more likely to choose conservative treatment.

In our country, the rehabilitation of most patients is usually conducted by the surgical clinic due to a lack of professional physical therapists. Consequently, the rehabilitation protocol would then depend on the surgeon and would generally tend to be more conservative (e.g. immobilisation for 8 to 12 weeks). In our study, the protocol provided to the patients was our recommendation. Whenever the patients were able to successfully perform single-legged heel raises, they were instructed to perform jogging 2 weeks later. Therefore, some patients might experience faster or slower recovery. Maffulli et al. [13] have reported that early functional rehabilitation and weight-bearing result in a shortening of rehabilitation time. Meanwhile, Mandelbaum et al. [17], who had used more sutures to strengthen the Achilles tendon, have shown that full weight-bearing is allowed in 2 to 3 weeks. In our study, partial weight-bearing while standing and moderate plantar flexion and dorsiflexion of the ankle were allowed just after the surgery for ATR. Even though the function of the ankle recovered rapidly without immobilisation after surgery, the patients might experience greater pain and risk of re-rupture in the early stage. Our findings revealed that group B (immobilisation for 2 weeks) experienced the optimal duration of immobilisation during the postoperative protocol, because they had a faster recovery with less pain than the other groups. However, all parameters were similar among the 4 groups after 16 weeks.

Minimally invasive surgery for ATR, such as limited open repair and percutaneous repair, was also alternative. Fortis et al. [22] reported that percutaneous repair with endoscopy was a safe technique with good outcomes and minimal complications, but potential problems included sural neuralgia and some decrease in strength. Henríquez et al. [21] reported that no significant differences were found between open and percutaneous repair. However, open repair with longitudinal posteromedial incision is still widely used, especially the Krackow suture [9]. In our study, the Krackow locking loop technique, combined with modified Kessler sutures, seemed to be a strong fixation for the ATR and allowed early weight-bearing and rehabilitation. Holm et al. [28] reported in a systematic review that re-rupture rates range from 2.0 to 10% after surgery. In comparison, our study reported a reduced re-rupture rate of 1.9% (5/266). We considered that due to our suture technique, early weight-bearing and rehabilitation could expand the patient’s ability to move the affected limb, which consequently increased their risk of accidents. We had noticed this after 5 patients who removed their brace, then experienced re-ruptures due to uncontrolled dorsiflexion. Therefore, after this study, if the patients needed to walk for long distances after removing the brace, such as going to work or travelling, we would advise them to wear a walking boot during their long walks until 8 weeks after surgery. Since then, we have completed this operation more than 200 times, and only one patient experienced early trauma-related re-rupture. We are still trying to find a better way to solve this problem.

In the cases without accidents, almost all patients experienced good recovery and minimal complications. Olsson et al. [29] reported a superficial wound infection rate of 12% after surgical treatment, whereas in our study, the infection rate was lower (1.9%, 5/266). Although the complication rate was higher in group A (5/42, 11.9%), no significant differences were found in complications among the 4 groups, which might be due to the small sample size of group A. During our study, we gradually realised that patients in group A might be more likely to experience complications, such as greater pain and risk of re-rupture. Therefore, we amended our recommendation, and no longer recommend that patients undergo no immobilisation period after surgery; consequently, only 42 patients were included in group A.

This study has some limitations. This was a retrospective cohort study, and although this is a large single-centre cohort study, the number of patients was relatively small. In addition, immobilisation time for each patient was selected in chronological order rather than by randomisation. Hence, we recommend a multicentre prospective randomised controlled trial to confirm our findings. Moreover, the sex discrepancy in our study may lead to sex bias. Additionally, no patient received any professional physical therapy, which could have affected the quality of their rehabilitation and led to improper wearing of the brace. Moreover, all clinical scores (VAS, AOFAS, and ATRS) with a subjective index might be inaccurate. Therefore, an objective physical score is needed to provide a more accurate evaluation of Achilles tendon functional recovery.

## Conclusion

Immobilisation for 2 weeks after open surgery for ATR is the best choice for early rehabilitation and weight-bearing while minimising pain and other complications.

## Data Availability

The datasets used and/or analysed during the current study are available from the corresponding author on reasonable request.

## Abbreviations

AOFAS: American Orthopaedic Foot and Ankle Society
ATR: Achilles tendon rupture
ATRS: Achilles tendon Total Rupture Score
DVT: Deep venous thrombosis
LSA: Light sports activity
ROM: Range of motion
SHRH: Single-legged heel rise height
SPSS: Statistical Package for the Social Sciences
VAS: Visual analogue scale

## References

[1] Houshian S, Tscherning T, Riegels-Nielsen P (1998). The epidemiology of Achilles tendon rupture in a Danish county. Injury..

[2] Maffulli N, Waterston SW, Squair J, Reaper J, Douglas AS (1999). Changing incidence of Achilles tendon rupture in Scotland: a 15-year study. Clin J Sport Med..

[3] Kangas J, Pajala A, Ohtonen P, Leppilahti J (2007). Achilles tendon elongation after rupture repair: a randomized comparison of 2 postoperative regimens. Am J Sports Med..

[4] Movin T, Ryberg A, McBride DJ, Maffulli N (2005). Acute rupture of the Achilles tendon. Foot Ankle Clin..

[5] Hess GW (2010). Achilles tendon rupture: a review of etiology, population, anatomy, risk factors, and injury prevention. Foot Ankle Spec..

[6] Khan RJ, Fick D, Brammar TJ, Crawford J, Parker MJ (2004). Interventions for treating acute Achilles tendon ruptures. Cochrane Database Syst Rev..

[7] Willits K, Amendola A, Bryant D, Mohtadi NG, Giffin JR, Fowler P, Kean CO, Kirkley A (2010). Operative versus nonoperative treatment of acute Achilles tendon ruptures: a multicenter randomized trial using accelerated functional rehabilitation. J Bone Joint Surg Am..

[8] Soroceanu A, Sidhwa F, Aarabi S, Kaufman A, Glazebrook M (2012). Surgical versus nonsurgical treatment of acute Achilles tendon rupture: a meta-analysis of randomized trials. J Bone Joint Surg Am..

[9] Kim U, Choi YS, Jang GC, Choi YR (2017). Early rehabilitation after open repair for patients with a rupture of the Achilles tendon. Injury..

[10] Gigante A, Moschini A, Verdenelli A, Del Torto M, Ulisse S, de Palma L (2008). Open versus percutaneous repair in the treatment of acute Achilles tendon rupture: a randomized prospective study. Knee Surg Sports Traumatol Arthrosc..

[11] Longo UG, Ronga M, Maffulli N (2009). Acute ruptures of the achilles tendon. Sports Med Arthrosc Rev..

[12] Keating JF, Will EM (2011). Operative versus non-operative treatment of acute rupture of tendo Achillis: a prospective randomised evaluation of functional outcome. J Bone Joint Surg Br..

[13] Maffulli N, Tallon C, Wong J, Lim KP, Bleakney R (2003). Early weightbearing and ankle mobilization after open repair of acute midsubstance tears of the achilles tendon. Am J Sports Med..

[14] Yotsumoto T, Miyamoto W, Uchio Y (2010). Novel approach to repair of acute achilles tendon rupture: early recovery without postoperative fixation or orthosis. Am J Sports Med..

[15] Kearney RS, Costa ML (2012). Current concepts in the rehabilitation of an acute rupture of the tendo Achillis. J Bone Joint Surg Br..

[16] Kangas J, Pajala A, Siira P, Hamalainen M, Leppilahti J (2003). Early functional treatment versus early immobilization in tension of the musculotendinous unit after Achilles rupture repair: a prospective, randomized, clinical study. J Trauma..

[17] Mandelbaum BR, Myerson MS, Forster R (1995). Achilles tendon ruptures. A new method of repair, early range of motion, and functional rehabilitation. Am J Sports Med..

[18] Bhandari M, Guyatt GH, Siddiqui F, Morrow F, Busse J, Leighton RK, Sprague S, Schemitsch EH (2002). Treatment of acute Achilles tendon ruptures: a systematic overview and metaanalysis. Clin Orthop Relat Res..

[19] Moller M, Movin T, Granhed H, Lind K, Faxen E, Karlsson J (2001). Acute rupture of tendon Achillis. A prospective randomised study of comparison between surgical and non-surgical treatment. J Bone Joint Surg Br..

[20] Deng S, Sun Z, Zhang C, Chen G, Li J (2017). Surgical treatment versus conservative management for acute Achilles tendon rupture: a systematic review and meta-analysis of randomized controlled trials. J Foot Ankle Surg..

[21] Henríquez H, Munoz R, Carcuro G, Bastias C (2012). Is percutaneous repair better than open repair in acute Achilles tendon rupture?. Clin Orthop Relat Res..

[22] Fortis AP, Dimas A, Lamprakis AA (2008). Repair of Achilles tendon rupture under endoscopic control. Arthroscopy..

[23] Carter TR, Fowler PJ, Blokker C (1992). Functional postoperative treatment of Achilles tendon repair. Am J Sports Med..

[24] Jaakkola JI, Beskin JL, Griffith LH, Cernansky G (2001). Early ankle motion after triple bundle technique repair vs. casting for acute Achilles tendon rupture. Foot Ankle Int..

[25] Kitaoka HB, Alexander IJ, Adelaar RS, Nunley JA, Myerson MS, Sanders M (1994). Clinical rating systems for the ankle-hindfoot, midfoot, hallux, and lesser toes. Foot Ankle Int..

[26] Nilsson-Helander K, Thomee R, Silbernagel KG, Thomee P, Faxen E, Eriksson BI (2007). The Achilles tendon Total Rupture Score (ATRS): development and validation. Am J Sports Med..

[27] Gwynne-Jones DP, Sims M, Handcock D (2011). Epidemiology and outcomes of acute Achilles tendon rupture with operative or nonoperative treatment using an identical functional bracing protocol. Foot Ankle Int..

[28] Holm C, Kjaer M, Eliasson P (2015). Achilles tendon rupture--treatment and complications: a systematic review. Scand J Med Sci Sports..

[29] Olsson N, Silbernagel KG, Eriksson BI, Sansone M, Brorsson A, Nilsson-Helander K, Karlsson J (2013). Stable surgical repair with accelerated rehabilitation versus nonsurgical treatment for acute Achilles tendon ruptures: a randomized controlled study. Am J Sports Med..

